# Sleep disorders in Parkinson’s disease

**DOI:** 10.1007/s00415-022-11403-5

**Published:** 2022-10-03

**Authors:** Jonathan Hawken, Neil Robertson

**Affiliations:** 1 Department of Neurology, University Hospital of Wales, Heath Park, Cardiff, CF14 4XN UK; 2 Department of Neurology, Division of Psychological Medicine and Clinical Neuroscience, Cardiff University, University Hospital of Wales, Heath Park, Cardiff, CF14 4XN UK

## Introduction

Parkinson’s Disease (PD) is a neurodegenerative disorder characterised by motor and non-motor features. Sleep disruption, including REM-sleep behaviour disorder and circadian rhythm dysfunction, is a well-recognised prodromal aspect of PD, although other features such as restless leg syndrome and other physical factors may contribute. Treatment of PD remains challenging due to the multifactorial nature of the disorder and requires a holistic approach. Improved understanding of central causes may be key to developing novel, effective and targeted treatments.

In this month’s journal club, we first review the association between alpha-synuclein in CSF and sleep disorders in prodromal and established PD. Secondly, we explore the role of Diffusion Tensor Imaging in assessing the glymphatic system in a similar cohort comparison. Finally, we will appraise the efficacy of Nabilone as a possible new therapeutic option for sleep disturbance in patients with established PD.

## Associations of sleep disorders with cerebrospinal fluid α-synuclein in prodromal and early Parkinson’s disease

Alpha-synuclein aggregation in brain tissue is a core pathological hallmark of PD. Routine testing of this protein in cerebrospinal fluid (CSF) is not currently established as a useful or necessary biomarker for diagnosis or monitoring of disease progression. However, recent studies have demonstrated lower CSF levels of alpha-synuclein in individuals with PD as compared to healthy controls. This study focusses on the relationship between sleep disorders and alpha-synuclein levels in CSF, comparing individuals with established PD, prodromal PD, and healthy controls. Sleep disturbance was measured by the REM-sleep Behaviour Disorder Screening Questionnaire (RBDSQ) and Epworth Sleepiness Scale (ESS).

575 patients were included in the study (360 individuals with PD, 46 with prodromal PD, and 169 healthy controls) with follow-up over a 3-year period. Individuals with prodromal PD were those with hyposmia (15/46) or REM-sleep behaviour disorder (RBD) (31/46). One-third of the prodromal PD group were included on the basis of hyposmia. This is a sensitive but not specific marker which may have biased results in this group.

At baseline, sleep disturbance by both measures was highest in the prodromal PD group, followed by the PD group, and healthy controls respectively. In keeping with previous research, CSF alpha-synuclein at baseline was lowest in the PD group, followed by the prodromal PD group, and then the control group. Participants with both PD and RBD-specific behaviours had significantly lower levels of CSF alpha-synuclein than those with only PD.

At follow-up, significantly decreased levels of CSF alpha-synuclein as compared to baseline were only seen in a subset of the patients (females with PD, prodromal PD patients specifically with dream-enactment behaviour, and healthy controls with daytime sleepiness).

**Comment:** Sleep disturbance is a prominent feature of PD and can predate the diagnosis by many years. RBD correlation with lower levels of CSF alpha-synuclein, as seen here in cases of prodromal and established, PD may provide an important window for treatment with future disease-modifying therapies targeted at halting or preventing alpha-synuclein aggregation.

Wang X. et al. (2022), Journal of Neurology, 269(5) 2469–2478. 10.1007/s00415-021-10812-2

## Neuroimaging evidence of glymphatic system dysfunction in possible REM sleep behaviour disorder and Parkinson’s disease

The glymphatic system has become a key focus of interest for neurodegenerative disorders. However, establishing a reliable imaging modality for assessment has proven challenging. Recently Diffusion Tensor Imaging (DTI) has been trialled in Alzheimer’s Disease, Normal Pressure Hydrocephalus and PD. This study used DTI along the perivascular space (DTI-ALPS) to evaluate glymphatic system activity in individuals with PD, RBD, and healthy controls. In limited studies of this technology to date, higher ALPS scores have been associated with an improved glymphatic activity.

A total of 416 patients were enrolled including 168 with PD, 119 with RBD, and 129 healthy controls at a single centre. Exclusion criteria of note included those with significant cerebral parenchymal or cerebrovascular abnormalities, very low cognitive scores, and subjects with poor-quality imaging. Age and gender were matched across the groups. However, significant differences were noted in multiple demographics including education, smoking, hypertension, cognitive and depression scores, and sleep assessment scores. Anti-parkinsonian medication was temporarily withheld prior to the MRI where relevant, though the effect of this on results is unclear. The Hoehn and Yahr scale identified a mean score of 2.34 within the PD cohort suggesting the exclusion of those with more advanced disease.

The ALPS index was lowest in individuals with PD, followed by RBD, and healthy controls respectively. ALPS scores also correlated with disease severity within the PD and RBD groups. A follow-up group of 50 patients with established PD did not show any significant difference in ALPS from their baseline scan, suggesting it may have limited value as a modality for monitoring disease progression, although the timeframe of expected change will require further investigation.

**Comment:** The novel application of this emerging technology, together with issues in cohort matching, limits applicability. However, results suggest that glymphatic system dysfunction is prominent in PD and RBD versus healthy controls and is detectable via DTI. Sleep disruption in PD may be one mechanism contributing to glymphatic dysfunction with sleep thought to enhance glymphatic activity. This aberrant process may result in alpha-synuclein accumulation and have relevance for disease progression.

Si X. et al. (2022), npj Parkinson's Disease, 8(1) 1–9. 10.1038/s41531-022-00316-9

## Effects of nabilone on sleep outcomes in patients with Parkinson’s disease: a post-hoc analysis of NMS-Nab study

Cannabinoids are gaining increasing attention for their possible therapeutic benefits. Nabilone is a synthetic cannabinoid currently used as an anti-emetic for symptoms related to cytotoxic chemotherapy. The NMS-Nab trial investigated the effects of Nabilone on non-motor symptoms (NMS) of Parkinson’s disease (PD). This paper describes the post-hoc analysis of the trial investigating the effects on sleep outcomes in those known to experience sleep problems at baseline.

This double-blind, placebo-controlled study was undertaken in a single centre in Austria. Inclusion criteria included age > 30 years, with stable motor symptoms and medication for > 30 days. Exclusion criteria included those with impulse control disorders, orthostatic hypotension, major psychiatric disorder, history of drug or alcohol abuse, and those with impaired liver function. Nabilone was given as an escalating dose regime up to a maximum of 2 mg, until NMS were much improved on the Clinical Global Impression of Improvement (CGI-I) scale. In phase 2, patients were randomised to continue on their optimal dose or placebo for 4 weeks. The medication was well tolerated with side effects consistent with its known profile.

In this post-hoc analysis, 31/38 patients randomised (14 Nabilone, 17 placebo) had sleep disturbance at baseline as measured by components of two analogous scales (NMSS and MDS-UPDRS-D2) and were compared. Following the initial treatment period, scores in both the MDS-UPDRS-1.7 and the NMSS-D2 improved. Of note, 96.8% of patients showed some improvement in MDS-UPDRS (≥ 1 point). A deterioration in score was seen in phase 2 in both groups. However, this was less in the Nabilone group. 29.7% of patients in the Nabilone group worsened by at least one point on the MDS-UPDRS suggesting a wearing-off effect may occur and a longer follow-up would be useful to explore this.

**Comment:** Nabilone demonstrated positive sleep effects in the majority of patients in this small study. Its widespread effects via the endocannabinoid system makes it challenging to determine in these individuals which symptoms (insomnia, pain, nocturia, or other) improved leading to this result. Caution is also needed in patient selection, in particular those with psychiatric disturbance or orthostatic dizziness and may limit its use in later stages of the disease.

Peball M. et al. (2022), Movement Disorders Clinical Practice, 9(6), 751–758. https://doi.org/10.1002/mdc3.13471

## Conclusion

These papers explore the important role of sleep in the progression and management of Parkinson’s disease. Levels of alpha-synuclein in CSF seem to correlate with REM-sleep behaviour disorder and this could suggest a time for therapeutic intervention. Glymphatic dysfunction leading to alpha-synuclein aggregation is an emerging area of interest but the role of imaging needs more validation. Nabilone demonstrated a positive short-term response in a majority of a small cohort of patients with PD and may prove a promising option in select patients.

